# Supplementary material for: B-Box Transcription Factor FaBBX22 Promotes Light-Induced Anthocyanin Accumulation in Strawberry (Fragaria × ananassa)
Source: Int J Mol Sci. 2022 Jul 14;23(14):7757. doi: 10.3390/ijms23147757 (PMC9316111; doi:10.3390/ijms23147757)
Supplement: Supplementary file 1 [file ijms-23-07757-s001.zip › Figure S1.pdf]

Figure S1. The coding sequences of FaBBX22

The sequence from translation start site (ATG) to stop codon for *FaBBX22* are shown as following.

>*FaBBX22*

ATGAAGATACAGTGTAACGTGTGCGAGGCGGCGGAGGCCACCGTGCTCTGCTGCGCC
GACGAGGCGGCGCTGTGCTGGGCCTGCGACGAGAAAGTCCACGCCGCCAACAAGCT
CGCCAGCAAGCACCAGAGGGTTCCTCTCTCCGCTTCTCATTTGCCCAAGTGCGATATT
TGCCAGGAGGCGGTTGGGTATTTCTTCTGCTTGGAGGATAGGGCTTTGCTATGTAGGA
AGTGCGATGTTGCGATACATACTGTGAATAGCTTTGTTTCCGGTCACCGGAGGTTTTTG
CTGACCGGAATTAAGGTTGGGCTGGAGCCCAATCCTCCGGGTAGGTCTGGTTCTGGTG
GCGGTGTTGCAGGGTCGTCTTCGTCGGTGAAATCGGAGTCTGGGGCCGTGGCGAAAT
GTGAGGGGAATAATCAGTTGGCTGAGGAATGCAAGGTGGCGCCGGAGAGTGTGGCC
GGGATGGGTTTTTCTGGGGGCTCTGGAGCTGGAGCTGGGTCTGTTCCGCAGTGGCCGA
TGGAGGAGTTTCTGCGATTCGCTGATTTCGATCAGAGCTTTGGATACATGGATAATGG
ATCGTCTAAGGCTGACTGCGGTAAACTTGGGGACTCTGATTCATCAGTTTTAAGATCA
CCTGAGGAGGAACTGGAGGATTACGAGTGCATAGGTCAGGTGCCAGAGCTCTCTTGG
ATGGTGCCACAGGTACCTTCCCCACCTACATCCTCAGGGCTATACTGGCCTAAAACTT
ATCAGAACCCATTGGATTCTGCGGTGTTTGTTCCAGACGTTAACTACTCAGATATGCA
AAATTATCTTCACTATCAACAGAATGGTACTGTCTCAAAACGTCGTAGGCAACTATA
A
